# Interactions of the Anti-SARS-CoV-2 Agents Molnupiravir and Nirmatrelvir/Paxlovid with Human Drug Transporters

**DOI:** 10.3390/ijms241411237

**Published:** 2023-07-08

**Authors:** Éva Bakos, Csilla Temesszentandrási-Ambrus, Csilla Özvegy-Laczka, Zsuzsanna Gáborik, Balázs Sarkadi, Ágnes Telbisz

**Affiliations:** 1 Research Centre for Natural Sciences—RCNS, Magyar Tudósok krt 2, 1117 Budapest, Hungary; bakos.eva@ttk.hu (É.B.); laczka.csilla@ttk.hu (C.Ö.-L.); sarkadi.balazs@ttk.hu (B.S.); 2 Charles River Laboratories, Irinyi József u. 4-20, 1117 Budapest, Hungary; csilla.temesszentandrasi-ambrus@crl.com (C.T.-A.); zsuzsanna.gaborik@crl.com (Z.G.)

**Keywords:** COVID-19, Nirmatrelvir, Paxlovid, Molnupiravir, ENT, CNT, OATP, ABC transporter

## Abstract

Orally administered small molecules may have important therapeutic potential in treating COVID-19 disease. The recently developed antiviral agents, Molnupiravir and Nirmatrelvir, have been reported to be efficient treatments, with only moderate side effects, especially when applied in the early phases of this disease. However, drug–drug and drug–transporter interactions have already been noted by the drug development companies and in the application notes. In the present work, we have studied some of the key human transporters interacting with these agents. The nucleoside analog Molnupiravir (EIDD-2801) and its main metabolite (EIDD-1931) were found to inhibit CNT1,2 in addition to the ENT1,2 nucleoside transporters; however, it did not significantly influence the relevant OATP transporters or the ABCC4 nucleoside efflux transporter. The active component of Paxlovid (PF-07321332, Nirmatrelvir) inhibited the function of several OATPs and of ABCB1 but did not affect ABCG2. However, significant inhibition was observed only at high concentrations of Nirmatrelvir and probably did not occur in vivo. Paxlovid, as used in the clinic, is a combination of Nirmatrelvir (viral protease inhibitor) and Ritonavir (a “booster” inhibitor of Nirmatrelvir metabolism). Ritonavir is known to inhibit several drug transporters; therefore, we have examined these compounds together, in relevant concentrations and ratios. No additional inhibitory effect of Nirmatrelvir was observed compared to the strong transporter inhibition caused by Ritonavir. Our current in vitro results should help to estimate the potential drug–drug interactions of these newly developed agents during COVID-19 treatment.

## 1. Introduction

The COVID-19 pandemic led to active vaccine development as well as the generation or re-purposing of small molecular compounds that potentially prevent SARS-CoV-2 infection and/or the development of a serious disease. Among these, Molnupiravir—a nucleoside analog—and Paxlovid were recently introduced in the clinic. Molnupiravir is a ribonucleoside analog [1] and Paxlovid is a combination of a specific virus protease inhibitor (Nirmatrelvir) and the “booster” Ritonavir, which slows down the metabolism of Nirmatrelvir [2]. Molnupiravir and Nirmatrelvir have received permission for short-term treatment of the early phases of the COVID-19 disease (see [3,4,5,6,7]). Currently, Molnupiravir has emergency use authorization from the FDA but permission for its use has been withdrawn by the EMA (see [3,4,5]). Paxlovid received approval from the FDA (U.S. Food and Drug Administration) and the EMA (European Medicines Agency) (see [6,7]). Both Molnupiravir and Nirmatrelvir showed strong antiviral effects in vitro [8,9] and were also effective under in vivo conditions [10,11,12,13,14].

Molnupiravir or EIDD-2801 is a prodrug that is rapidly converted to EIDD-1931 in the blood plasma and the potent antiviral agent is the 5′-triphosphate of EIDD-1931, generated by host intracellular kinases [1,8,15]. Nirmatrelvir (PF-07321332) was developed as an inhibitor of the key protease (Mpro, 3Clpro) in coronaviruses [10,16] and adjusted for better oral bioavailability [17,18]. In Paxlovid, Ritonavir is applied to enhance the tissue availability of Nirmatrelvir by inhibiting the CYP3A4 enzyme involved in the rapid metabolism of Nirmatrelvir [10], although this combination may significantly increase drug–drug interactions (DDIs) either by CYP3A4 or drug-transporter-related mechanisms [2,19,20].

In the present work, our aim was to reveal potential DDI effects by identifying selected drug–transporter interactions of Molnupiravir, Nirmatrelvir, and the Nirmatrelvir/Ritonavir combination with a set of drug transporters. We focused on studying key cellular drug uptake transporters in the Solute carrier family (SLC): Organic anion transporting polypeptides; OATPs, namely OATP1A2/SLC21A3, OATP1B1/SLC21A6, OATP1B3/SLC21A8, OATP2B1/SLC21A9, ENT1/SLC29A1 (Equilibrative nucleoside transporter 1), ENT2/SLC29A2, CNT1/SLC28A1 (Concentrative nucleoside transporter 1), and CNT2/SLC28A2. We also investigated the major drug extruders in the ATP Binding Cassette (ABC) family, multidrug resistance protein ABCB1/P-gp/MDR1 (Multidrug resistance protein 1), ABCG2/BCRP (Breast cancer resistance protein), and MRP4/ABCC4 (Multidrug resistance-related protein 4). SLC and ABC transporters of clinical relevance are known to potentially alter the pharmacokinetics of drugs since they are expressed at important tissue barriers such as the intestine or the blood–brain barrier [21]. In the case of the nucleoside analog Molnupiravir, we included in these studies the main human nucleoside transporters (ENT1, ENT2, CNT1, CNT2, and MRP4). Our in vitro studies should provide valuable information on in vivo drug–transporter and drug–drug interactions that are potentially associated with the clinical side effects of these widely used anti-COVID-19 agents.

## 2. Results

### 2.1. Interactions of Molnupiravir with Selected Human Membrane Transporters

We have focused on the examination of the effects of the nucleoside analog Molnupiravir (EIDD-2801) and its metabolite (EIDD-1931) found in the blood plasma on the function of selected human membrane transporters involved either in nucleoside transport or multi-specific drug transport. When examining the effects of EIDD-2801 and EIDD-1931, we studied a wide range of drug concentrations, as the respective transporters may interact with highly variable drug concentrations at the site of intestinal absorption or drug excretion.

**Figure 1:**
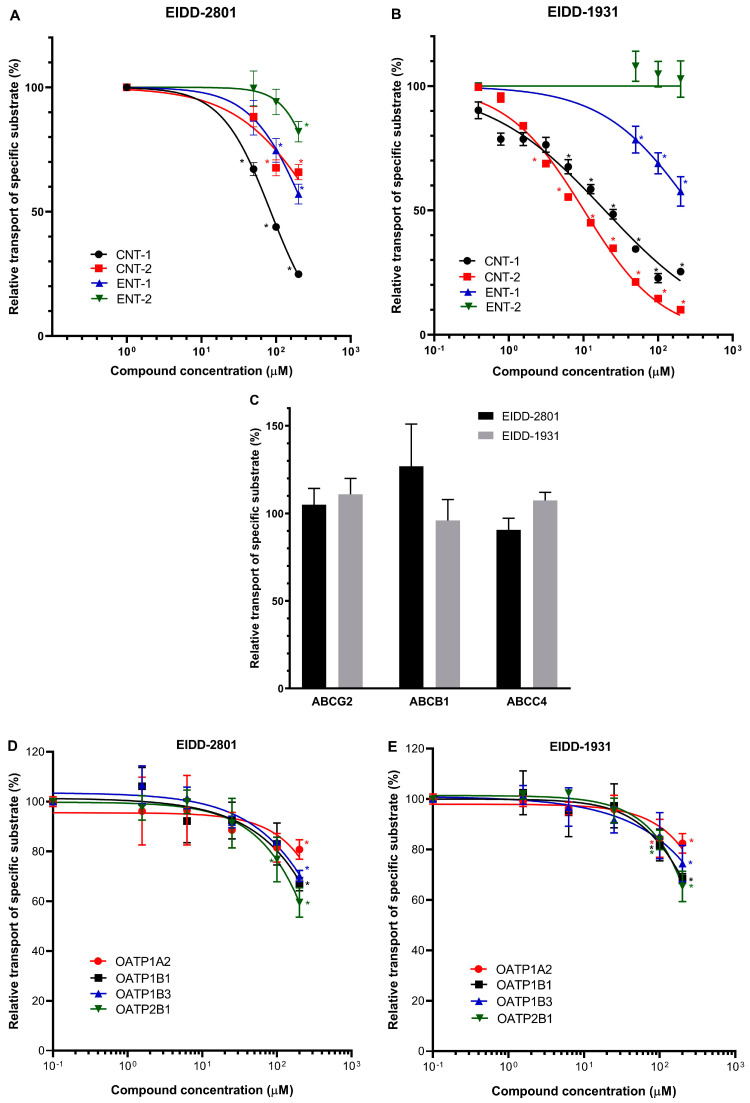
Effects of Molnupiravir (EIDD-2801) and its derivative (EIDD-1931) on the function of transporters. Panel (**A**,**B**). Uridine uptake was measured in CNT1-MDCKII, CNT2-MDCKII, and ENT1-MDCKII cells. Adenosine uptake was measured in ENT2-MDCKII cells. Panel (**C**). Transport activity of ABC transporters was measured in inverted membrane vesicles prepared from overexpressing HEK293 cells. Transport of specific substrates (LY, NMQ, and DHEAS) was measured using ABCG2, ABCB1, and ABCC4, respectively. Relative substrate transport in the presence of 200 μM of Molnupiravir or EIDD-1931 is shown. Panel (**D**,**E**). OATP activity was measured in transporter-overexpressing A431 cells using pyranine (OATP1B1, OATP1B3, OATP2B1) or SR101 (OATP1A2) as test substrates. Mean ± SEM of 3 independent experiments are shown. Significant differences (*p* < 0.01) between test and control values are labeled with asterisks.

The key human cellular nucleoside uptake transporters are CNT1 and CNT2 (SLC28A1, SLC28A2) and ENT1 and ENT2 (SLC29A1, SLC29A2) [22,23]. Highly variable inhibitory effects of either EIDD-2801 or EIDD-1931 were observed in MDCKII cells overexpressing any of these uptake transporters (Figure 1). CNT1-mediated uridine uptake was potently inhibited by both EIDD-2801 (IC_50_ = 84.89 µM, 95% CI 75.13–95.17 µM and EIDD-1931 (IC_50_ 18.77 µM, 95% CI 14.15–24.97 µM), reaching approximately 80% inhibition at 200 μM concentration. In contrast, CNT2-mediated uridine uptake was less potently inhibited by EIDD-2801, while a strong inhibition of this transporter was observed when the active metabolite, EIDD-1931 (IC_50_ = 10.17 µM, 95% CI 8.42–12.3 µM) (Figure 1A,B), was used.

In ENT-type nucleoside uptake transporter studies, ENT1-mediated uridine transport was inhibited to about 50% of the full activity by both EIDD-2801 and EIDD-1931 at 200 μM. Interestingly, uptake of the specific substrate (adenosine) by ENT2 was only mildly inhibited by EIDD-2801 compound (Figure 1A,B).

ABCC4 (MRP4) has been reported to function as a key cellular nucleoside exporter [24,25,26]. In our experiments, we measured the effects of Molnupiravir (EIDD-2801) and its derivative (EIDD-1931) on the function of the ABCC4 nucleoside transporter in inverted membrane vesicles prepared from ABCC4-overexpressing HEK cells. In these studies, ATP-dependent DHEAS uptake into the membrane vesicles was measured and MK-571 was used as a specific transporter inhibitor. When examining either EIDD-2801 or EIDD-1931 in concentrations up to 200 μM, we found no significant effects on the activity of the MRP4 transporter (Figure 1C).

Transport activities of the key multidrug exporters (ABCB1 and ABCG2) were also investigated in the presence of EIDD-2801 and EIDD-1931. These experiments were performed using inverted membrane vesicles prepared from transporter-overexpressing cells and ATP-dependent uptake of N-methyl-quinidine (NMQ) for ABCB1 and lucifer yellow (LY) transport into ABCG2-containing membrane vesicles, were measured. As shown in Figure 1C, neither the function of ABCB1 nor that of ABCG2 was altered either by Molnupiravir or its derivative EIDD-1931. In subsequent experiments, we examined the effects of EIDD-2801 and EIDD-1931 on the function of OATP-type multidrug uptake transporters. In each case, transporter-overexpressing cells and specific uptake transporter substrates were applied in these studies, i.e. pyranine for OATP1B1, OATP1B3, OATP2B1, and SR101 for OATP1A2. Neither EIDD-2801 nor EIDD-1931 had a major effect on the specific activity of the OATP1A2, OATP1B1, OATP1B3, or OATP2B1 transporters (Figure 1D,E). Only weak (maximum of 20–40%) inhibitions were observed at 200 μM concentrations of EIDD-2801 or EIDD-1931. IC50 values could not be determined, but our estimation is more than 200 μM.

### 2.2. Interactions of Nirmatrelvir (PF-07321332) and Nirmatrelvir–Ritonavir Combination with Selected Drug Transporters

**Figure 2:**
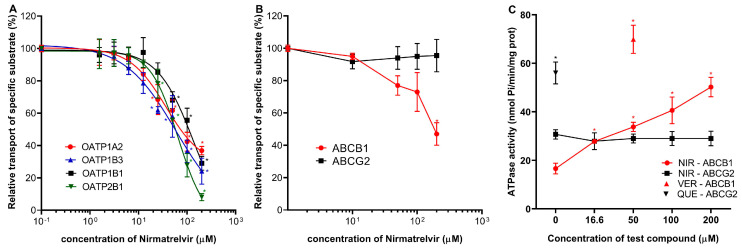
Effects of Nirmatrelvir on OATP uptake transporters and ABC multidrug transporters. Panel (**A**): Activity of OATP1A2, OATP1B1, OATP1B3, and OATP2B1 transporters were examined using pyranine (for 1B1, 2B1, and 1B3) and SR101 (for 1A2) as test substrates in transporter-overexpressing A431 cells. Transport is shown in the presence of increasing concentrations of Nirmatrelvir. Panel (**B**): Influence of Nirmatrelvir on the transport activity of ABCB1 (NMQ transport) and ABCG2 (LY transport) in inverted membrane vesicles prepared from transporter-overexpressing HEK cells. Panel (**C**): Effect of Nirmatrelvir (NIR) on the ATPase activity of ABCB1 and ABCG2 measured in inverted membrane vesicles prepared from transporter-overexpressing insect cells. As a reference, a known stimulatory substrate for each transporter is presented on the panel at its maximally effective concentration. These are Verapamil (VER, 50 μM) for ABCB1 and Quercetin (QUE, 1 μM) for ABCG2. Mean ± SEM of 3 independent experiments are shown. Significant differences (*p* < 0.01) between test and control values are labeled with asterisks.

The most important drug–transporter interactions for PF-07321332 (Nirmatrelvir) have already been reported, and the influence of Ritonavir on the pharmacokinetic parameters of Nirmatrelvir has been characterized (as in Paxlovid treatment) [2,19]. Here we examined the specific effect of Nirmatrelvir and its combination with Ritonavir on selected drug transporters that have not been examined in previous in vitro approaches. Regarding the uptake transporters, we found that Nirmatrelvir showed significant inhibitory potential against specific substrate transport by OATP1A2, OATP1B1, OATP1B3, and OATP2B1 (Figure 2A), although 50 μM or higher concentrations of Nirmatrelvir were needed to achieve 50% OATP inhibition. Estimated IC_50_ values were 79.5 μM (95% CI 96.5–110.6 µM) for OATP1A2, 109.8 μM (95% CI 86.35–137.8 µM) for OATP1B1, 56.2 μM (39.6–77.4 µM) for OATP1B3, and 58.7 μM (95% CI 55–62.7 µM) for OATP2B1. Following the recent M12 DDI risk evaluation recommendation, the calculated Cmax,inlet,u/OATP1B1 IC_50_ and Cmax,inlet,u/OATP1B3 IC_50_ ratios were 0.41 and 0.78. The ratios were slightly above the cut-off value of 0.1.

In vesicular transport assays, the transport of lucifer yellow by ABCG2 was not affected by up to 200 μM concentrations of Nirmatrelvir. Conversely, Nirmatrelvir had a moderate inhibitory effect on ABCB1. According to the M12 guideline, we calculated (dose (300 mg)/250 mL)/IC_50_ ratio. Although the estimated IC_50_ value is high for approximately 200 µM (precise determination is not possible for such high concentrations), the calculated ratio is 12, slightly above the cut-off value of 10 for ABCB1 (Figure 2B). Interestingly, the transport-coupled ATPase activity of the ABCB1 protein was significantly increased by Nirmatrelvir, while there was no such effect of Nirmatrelvir on the ATPase activity of the ABCG2 transporter (Figure 2C).

In subsequent experiments, we examined the combined effects of Nirmatrelvir and Ritonavir to simulate the possible effects of Paxlovid treatment on the transporters. The recommended clinical dose of Paxlovid is 300 mg Nirmatrelvir and 100 mg Ritonavir [18]. Corresponding to this dose ratio and the observed plasma concentrations (5 μM Nirmatrelvir and 0.5 μM Ritonavir), ratios of 2/1 (reflecting intestinal ratios) and 10/1 (plasma ratios) were used in these experiments [10].

**Figure 3:**
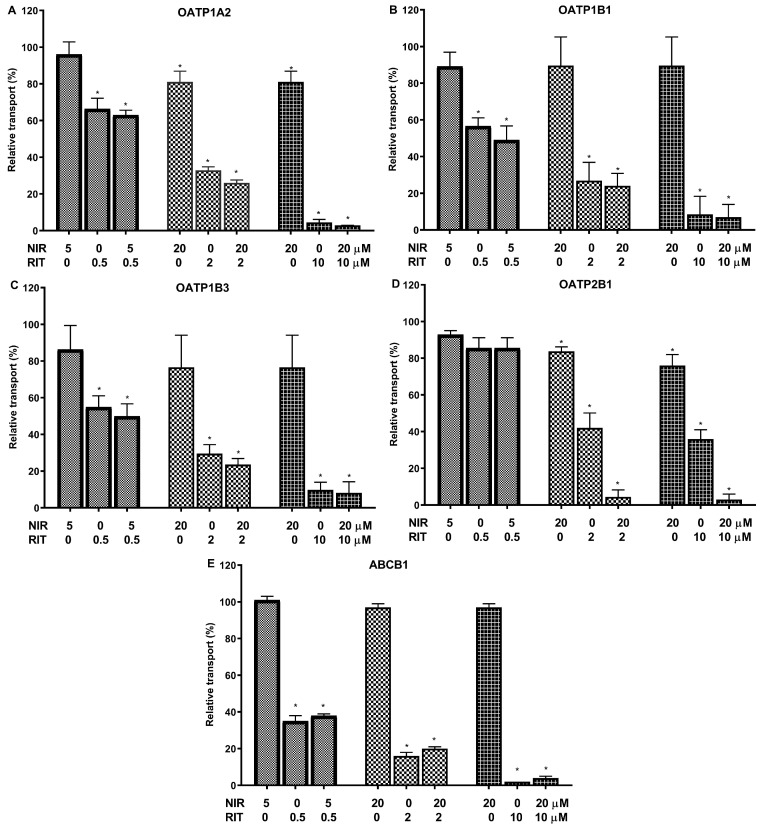
Combined effects of Nirmatrelvir (NIR) and Ritonavir (RIT) on the transport activities of OATP1A2 (Panel (**A**)), OATP1B1 (Panel (**B**)), OATP1B3 (Panel (**C**)), OATP2B1 (Panel (**D**)), and ABCB1 (Panel (**E**)). The functions of the OATP1A2, OATP1B1, OATP1B3, and OATP2B1 transporters were examined using pyranine (for 1B1, 2B1, and 1B3) and SR101 (for 1A2) as test substrates in transporter-overexpressing A431 cells, while NMQ transport by ABCB1 was examined in HEK293 cell membrane vesicles. Transport was measured in the presence of Nirmatrelvir alone or in combination with ritonavir at the indicated concentrations. Mean ± SEM of 3 independent experiments are shown. Significant differences (*p* < 0.01) between test and control values are labeled with asterisks.

As shown in Figure 3A–D, Nirmatrelvir alone had only a minor inhibitory effect on OATP1A2, OATP1B1, OATP1B3, and OATP2B1 transporter functions at 20 μM, the maximal concentration applied in combination therapies. The 20 μM NIR/10 μM RIT concentrations had near maximum inhibition of all OATPs examined, and even 5 μM NIR/0.5 RIT μM drug concentrations caused about 50% transporter inhibition. However, this inhibitory effect was clearly due to the presence of Ritonavir, and Nirmatrelvir had only a negligible additive effect in most cases.

Similar results were obtained for the ABCB1 transport activity (Figure 3E). In this case, Ritonavir also caused a strong inhibition of the ABCB1 function even at low (0.5 μM) concentrations, but no additional effect of Nirmatrelvir was observed.

## 3. Discussion

In this study, we have characterized two recently developed and clinically recommended anti-COVID-19 agents, Molnupiravir and Paxlovid, in terms of their interactions with important membrane transporters. Considering the role of multi-specific drug transporters in the absorption, tissue distribution, excretion, and toxicity (ADME-tox properties) of their substrate drugs [21], their importance may be particularly relevant in the treatment of severe COVID-19 patients, many of whom have underlying health problems or diseases that are already being treated with drugs or drug combinations.

We studied potentially relevant transporter interactions of Molnupiravir and Paxlovid. In the present work, we used well-established in vitro assays, most of which are widely applied in drug development, to study the functions of specific transporter proteins. We focused on multi-specific drug transporters that may be relevant to the ADME-tox and DDI effects of these drugs.

The clinically active form of Molnupiravir (EIDD-2801) is formed in the blood plasma where it is converted to EIDD-1931, which strongly inhibits viral RNA polymerase and has only negligible toxicity in human cells [27]. The recommended dose of Molnupiravir for COVID-19 treatment is 800 mg, given twice a day. At this dose, the peak plasma concentration of EIDD-1931 was found to be 2–3 μg/mL, less than 10 μM [1,18].

In our experiment, we examined the transporter interactions of both EIDD-2801 and EIDD-1931 because both forms can interact with transporters at tissue barriers. The key human nucleoside transporters studied were CNT1 and CNT2 (SLC28A1, SLC28A2), as well as ENT1 and ENT2 (SLC29A1, SLC29A2). These transporters are multi-specific in their recognition of nucleosides and are also involved in antiviral drug resistance [24,25,28,29].

We found that the transport functions of ENT1 and ENT2 were not significantly inhibited by Molnupiravir and its derivative at the low concentrations available in the plasma, although inhibition may occur at high concentrations. These results are in good agreement with published in vitro data [30]. It is known that ENT and CNT can transport and modulate the distribution of many nucleoside-type drugs. Nevertheless, significant clinical side effects and DDIs that may be associated with ENT or CNT transporters are not indicated. Therefore, there is currently no unified recommendation to calculate the relevance of in vitro effects for in vivo application in this context. It is believed that the high IC_50_ Molnupiravir values do not indicate a significant risk of DDI from ENT or CNT. A recent article summarized some of the clinically relevant DDIs of ENT and CNT inhibitors, some of which may also be considered for Molnupiravir therapy [31]. The activity of the ABCC4 nucleoside exporter was not modified by EIDDs. When we examined the interactions between EIDD-2801 and EIDD-1931 and the OATP uptake transporters (OATP1A2, OATP1B1, OATP1B3, OATP2B1) and the ABCB1 and ABCG2 exporters, we again found no conspicuous inhibitory effects. Regarding potential plasma concentrations of the Molnupiravir derivative EIDD-1931 during COVID-19 therapy, our in vitro studies suggest no significant in vivo DDIs for multidrug transporters. Even at specific places where Molnupiravir concentrations are higher, for example, the intestines or liver, DDI effects are unlikely.

Nirmatrelvir (PF-07321332) showed antiviral activity in the nanomolar range against SARS-CoV-2 in cell culture tests and had similar effectiveness against various coronaviruses [10]. Nirmatrelvir was shown to be effective against various SARS-CoV-2 variants (alfa, delta, various types of omicron) in cell culture tests [32]. This was also confirmed in human studies where patients infected with different omicron variants were treated with Paxlovid and followed in a clinical study (USA-WA1/2020) [33]. It appears that short-term use of Paxlovid increased adverse events by only a few percent, although adverse events in high-risk patients are not easy to track because of cumulative health problems [34]. In the case of orally applicable Nirmatrelvir, particularly in the combination form of Paxlovid, data in the literature and the drug application notes indicate numerous potential drug interactions [19,35,36]. In the Paxlovid combination, Ritonavir is used to slow down the metabolism of Nirmatrelvir by inhibiting its metabolism by CYP3A4 [2,10]. However, as previously noted, Ritonavir is also a strong inhibitor of numerous drug transporters [28,37,38,39], thus potential interactions with Ritonavir should be considered.

The recommended clinical dose of Paxlovid is 300 mg Nirmatrelvir and 100 mg Ritonavir, administered twice daily. In this case, the plasma peak concentration of NIR is around 2 μg/mL (appr. 4–5 μM) [18]. When we examined the various uptake transporters, we found that Nirmatrelvir had a relatively small effect on substrate transport by OATP1A2, OATP1B1, OATP1B3, and OATP2B1. These data are consistent with those published by the Pfizer research group on OATP1B1 and OATP1B3, where the IC_50_ of OATP1B1 was 44 μM for rosuvastatin transport and the IC_50_ for OATP1B3 was more than 100 μM [2]. Slightly different IC_50_ values (110 μM for OATP1B1 and 59 μM for OATP1B3) were obtained in our investigation of other substrates. Regarding the multi-specific drug exporters and in agreement with previous findings [2,10], only a weak inhibitory effect of Nirmatrelvir was observed against ABCB1, and a negligible effect was observed against the ABCG2 transporter. Nirmatrelvir stimulated the substrate-dependent ATPase activity of ABCB1 in isolated membrane vesicles, suggesting that Nirmatrelvir is a substrate transported by this protein. This result is consistent with the ABCB1-mediated transport of Nirmatrelvir observed in studies of the MDCKII monolayer [2]. Because Nirmatrelvir is an ABCB1 substrate, ABCB1 is likely to modulate the absorption and tissue distribution of this compound, but in vivo, CYP3A4-mediated metabolism is considered to be the major factor in the pharmacokinetics of Nirmatrelvir [2]. The calculated IC_50_ values for OATP transporters and ABCB1 exceed the Cmax value for NIR by two orders of magnitude. Due to this difference, comprehensive DDIs cannot be postulated. However, the current assessment of the DDI risk is based on the M12 recommendation (available for ABCB1, OATP1B1, and OATP1B3), which uses much higher estimated drug concentrations in the liver and intestines. According to this recommendation, the extrapolated risk values are slightly higher than the cut-off values for nirmatrelvir with ABCB1, OATP1B1, and OATP1B3, hence potential risk cannot be excluded, and in vivo, drug interaction studies are required. There is no recommendation on how to calculate the in vivo relevance for OATP1A2 and OATP2B1, but high IC_50_ values do not suggest a significant clinical DDI problem. In contrast to the data above, which show only weak inhibition of the investigated transporters by Nirmatrelvir, greater effects on drug transporters were observed with combinations of Nirmatrelvir and Ritonavir, used in our experiments in appropriate ratios as in Paxlovid. A strong inhibitory effect of Ritonavir on numerous drug transporters (OATPs, ABC transporters) is well known, as summarized in our recent studies [28,37]. Although we found little or no additive effects of Nirmatrelvir on Ritonavir, this drug combination should be considered in comedications as a potent inhibitor of numerous multidrug transporters, including various OATPs, ABCB1, and ABCG2, and not only as an inhibitor of the CYP enzymes [2,37].

## 4. Materials and Methods

### 4.1. Materials

EIDD-1931, EIDD2801, and Nirmatrelvir (PF-073213332) were purchased from MedChemExpress (Shanghai, China). Stock solutions of the compounds were freshly prepared in dimethyl sulfoxide (DMSO). Non-radiolabeled chemicals were obtained from Merck/Sigma-Aldrich (St. Louis, MO, USA). All chemicals were of analytical grade. ^3^H-Uridine ([5-^3^H], 20.9 Ci/mmol), and ^3^H-Adenosine ([2,8-^3^H], 29.3 Ci/mmol) were purchased from Moravek Biochemicals Inc. (Brea, CA, USA). Ultima Gold XR scintillation fluid was purchased from PerkinElmer (Waltham, MA, USA).

### 4.2. ENT and CNT Transporter Uptake Assays

ENT and CNT transporter interactions were investigated in transporter-overexpressing cell lines [40]. Madin-Darby Canine Kidney II (MDCKII) cell lines overexpressing the human uptake transporters CNT1, CNT2, ENT1, and ENT2 were generated via lentiviral transduction. Cell cultures were maintained in Dulbecco’s modified Eagle’s medium (DMEM) and 4500 mg/L of glucose supplemented with GlutaMax^TM^, 10% *v*/*v* fetal bovine serum (FBS), 100 units/mL penicillin, and 100 µg/mL streptomycin (all from Gibco/ThermoFisher, Waltham, MA, USA) at 37 °C in 5% CO_2_ at 95% humidity. For uptake inhibition assays, transporter-expressing cells and mock-transduced control cells were seeded on 96-well tissue culture plates at a density of 1 × 10^5^ cells/well. Twenty-four hours after seeding, cells were rinsed twice with Krebs-Henseleit buffer (KH buffer, pH 7.4) and then preincubated for 30 min with solutions containing serial dilutions of the test compounds in KH at room temperature. Following preincubation, the solutions were removed, the cells were rinsed twice with KH buffer, and coincubation was started with a suitable radiolabeled substrate and the diluted series of test compounds. Assay conditions were as follows: 1 µM uridine (traced with 0.1 µCi ^3^H-Uridine/well), 30 s (CNT1), and 1 min (CNT2, ENT1) incubations; 1 µM adenosine (traced with 0.1 µCi ^3^H-Adenosine/well) and 2 min (ENT2) incubation. All experiments were conducted at room temperature. The concentration of the organic solvent was equal in all wells and did not exceed 1.5% (*v*/*v*). Uptake was terminated by rinsing twice with ice-cold KH buffer, and cells were lysed with 0.1 M NaOH. To determine the amounts of accumulated radiolabeled substrates, cell lysates mixed with a liquid scintillation cocktail were measured with a MicroBeta2 microplate counter (PerkinElmer, Singapore).

Transporter-specific accumulation of the probe substrate was calculated using the following formula: ACC_Spec_ = ACC_TRP_ − ACC_Mock_, where ACC_TRP_ is the accumulation of the probe in transporter-expressing cells and ACC_Mock_ is the accumulation of the probe in control cells. Relative transporter-specific accumulation values were calculated as percentages of the control value (with vehicle only).

### 4.3. Transport and ATPase Measurements of ABC Transporters in Membrane Vesicles

ABCB1, ABCG2, and ABCC4 HEK293 membrane vesicles were prepared by Solvo Biotechnology, Budapest. The total protein content of membrane vesicle preparations was determined using the Lowry quantitation method and 30 µg (ABCG2) or 50 µg (ABCB1 and ABCC4) total protein/sample was used for the measurements. For ABCG2 vesicular transport, the fluorescent probe Lucifer yellow (LY) was present in the mixture at 5 μM final concentration. Radiolabeled N-methyl quinidine (NMQ, 1 μM) and radiolabeled dehydroepiandrosterone sulfate (DHEAS, 0.5 µM) substrates were used for ABCB1 and ABCC4, respectively. Uptake of the probe substrates into the inverted membrane vesicles was measured at 37 °C (ABCG2, ABCB1) or at 32 °C (ABCC4) for 10 min (ABCG2), 5 min (ABCB1), or 1.5 min (ABCC4) in the presence or absence of 4 mM Mg-ATP, in 75 μL final volume of the media (0.04 M MOPS-TRIS pH 7.2, 56 mM KCl, 6 mM MgCl_2_). The known reference inhibitors of the transporters (1 µM Ko143 for ABCG2, 50 µM Verapamil for ABCB1, and 150 µM MK571 for ABCC4) served as controls. Each test compound was dissolved in DMSO and a 0.75 µL volume was added to the samples. A DMSO solvent control was used in each experiment. The transport was stopped by the addition of ice-cold wash buffer to the samples, then rapidly filtered (MSFBN6B, Millipore, Burlington, MA, USA). Filters were washed with ice-cold wash buffer (0.04 M MOPS-TRIS pH 7.2, 70 mM KCl). For LY, vesicles retained on the filters were dissolved in 10% SDS and transferred into a 96-well microplate. After the addition of DMSO as a stabilizer, fluorescence was measured using a plate reader at 427/535 nm (Victor X3 Enspire Perkin-Elmer, Waltham, MA, USA). When radiolabeled compounds were used as substrates, radioactivity retained on the filter was measured by liquid scintillation counting in Optiphase HiSafe liquid (Perkin Elmer MicroBeta2 liquid scintillation counter, Perkin Elmer, Waltham, MA, USA). ATP-dependent transport was calculated by subtracting the values obtained in the presence of AMP from those obtained in the presence of ATP.

ATPase activity was measured in Sf9 membrane vesicles containing the respective human ABC transporters, as described in our earlier work [41]. For ABCG2, the cholesterol level in the vesicles was increased to the level of mammalian cell membranes to obtain full activity [42]. In brief, membrane vesicles (5 μg protein/sample) were incubated with 3 mM Mg-ATP and various concentrations of test compounds. Background ATPase activity of membranes was determined by applying the general ABC transporter inhibitor sodium orthovanadate (1 mM). Measurements were carried out for 20 min at 37 °C. The amount of phosphate liberated from ATP was detected using a colorimetric method [41] and a VictorX3 plate reader at 660 nm Abs. Reference activators (50 µM verapamil for ABCB1 and 1 µM quercetin for ABCG2) served as positive controls.

### 4.4. OATP Uptake Assay

To study the function of OATPs, we used A431 cells overexpressing OATP1A2, OATP1B1, OATP1B3, or OATP2B1 and mock-transfected A431 cells, generated as previously described [43,44], as controls. The interactions between OATPs and the test compounds were studied in a microplate-based indirect assay by employing fluorescent dye substrates: pyranine for OATP1B1, OATP1B3, OATP2B1 and sulforhodamine 101 for OATP1A2 [44,45]. Briefly, OATP-overexpressing A431 cells and mock-transfected A431 cells were seeded on 96-well plates (8 × 10^4^ cells/well). The next day, the cells were washed three times with phosphate buffered saline (PBS, pH 7.4) and preincubated with 50 μL uptake buffer (125 mM NaCl, 4.8 mM KCl, 1.2 mM CaCl_2_, 1.2 mM KH_2_PO_4_, 12 mM MgSO_4_, 25 mM MES (2-(Nmorpholino) ethanesulfonic acid, and 5.6 mM glucose, pH 5.5 for OATP2B1 and pH 7.4 for OATP1A2, OATP1B1, or OATP1B3) at 37 °C for 5 min in the presence or absence of the tested compounds. The reaction was started by the addition of a final concentration of 20 μM pyranine (OATP1B1, OATP1B3, OATP2B1) or 0.5 μM sulforhodamine 101 (OATP1A2). After incubation at 37 °C for 10 min, the reactions were stopped and the cells were washed three times with ice-cold PBS. Fluorescence was measured using an Enspire plate reader (Perkin Elmer, Waltham, MA, USA) (Ex/Em: 460/510 nm (pyranine) or 586/605 nm (sulforhodamine 101), and OATP-dependent transport activity was determined by subtracting the fluorescence measured in mock-transfected cells.

### 4.5. Data and Statistical Analysis

Microsoft Excel 365 (Microsoft Corporation, Redmond, WA, USA) was used for basic data processing and GraphPad Prism 9.4.1 (GraphPad Software Inc., San Diego, CA, USA) was used for determination of inhibition potencies.

For the estimation of potential in vivo drug effects, calculations described in ICH Harmonized Guideline Drug Interaction Studies M12 and available pharmacokinetic data for the compounds were used. The clinically recommended dosage is 300 mg Nirmatrelvir (two 150 mg tablets) with 100 mg Ritonavir (see [18]). (Dose/250 mL)/ABCB1 IC_50_ ratio was calculated for Nirmatrelvir. The estimated unbound maximum plasma concentration of Nirmatrelvir at the liver inlet (Cmax,inlet,u) was calculated and was equal to Cmax + (ka × Dose × FaFg)/Qh/RB, where Cmax is the maximum systemic plasma concentration of Nirmatrelvir; ka is the absorption rate constant. ka = 0.1/min was used as the worst-case estimate; Fa is the fraction absorbed, and Fa = 1 was used as the worst-case estimate. Fg is the intestinal availability and Fg = 1 was used as the worst-case estimate; Qh is the hepatic blood flow rate. Qh = 1450 mL/min was used; RB is the blood-to-plasma concentration ratio, assumed to be 1. Then, Cmax,inlet,u/OATP1B1 IC_50_ and Cmax,inlet,u/OATP1B3 IC_50_ ratios were calculated.

## Data Availability

Data is contained within the article. Other data will be available on request within 6 months of this publication via direct communication with the corresponding author.
